# Telehealth Utilization and Patient Experiences: Patterns of Social Determinants of Health Among Individuals With Hypertension and Diabetes

**DOI:** 10.1016/j.jacadv.2026.102888

**Published:** 2026-07-22

**Authors:** Haoxin Chen, Will Simmons, Malak Abu Hashish, Jiancheng Ye

**Affiliations:** a The University of Hong Kong, Hong Kong, China; b Weill Cornell Medicine, Cornell University, New York, New York, USA; c Touro University College of Osteopathic Medicine, Great Falls, Montana, USA; d Feinberg School of Medicine, Northwestern University, Chicago, Illinois, USA

**Keywords:** diabetes, hypertension, multiple chronic conditions, social determinants of health, technology utilization, telehealth

## Abstract

**Background:**

Telehealth has become a critical modality for managing chronic diseases like hypertension and diabetes. Understanding utilization patterns, effectiveness, and how social determinants of health (SDOH) influence access is essential for promoting equitable care in diverse populations.

**Objectives:**

The purpose of this study was to evaluate telehealth utilization, effectiveness, and patient satisfaction among individuals with hypertension and/or diabetes, and examine how SDOH relate to telehealth access across sociodemographic groups in the United States.

**Methods:**

We conducted a cross-sectional analysis of the 2022 Health Information National Trends Survey 6. Telehealth use was measured across 14 survey items. Participants were categorized as having diabetes only, hypertension only, or both. Weighted analyses used Taylor series methods with appropriate statistical tests and multiple comparison adjustments.

**Results:**

Of 6,252 respondents, 3,009 met inclusion criteria. Overall telehealth utilization was 43.9%. Participants with both conditions were significantly older, more likely to be retired, and had lower income and education compared to single-condition groups. Telehealth modality differed across groups: the comorbid group had the highest phone-only use (21.4%), while the diabetes-only group had the highest video-only use (21.4%). Telehealth was offered to only 13.2% to 21.5% of participants. No significant differences were found in technical difficulties, perceived care quality, or privacy concerns. SDOH challenges including food insecurity, transportation barriers, and lower education were most concentrated in the comorbid group.

**Conclusions:**

Despite bearing the greatest SDOH burden, the comorbid group showed the highest utilization, likely reflecting care burden rather than equitable access, reinforced by disproportionate reliance on phone-only visits. Targeted interventions addressing digital access and SDOH barriers are needed to ensure equitable telehealth delivery.

Chronic diseases such as hypertension and diabetes are prevalent health concerns that significantly impact individuals’ quality of life and pose substantial challenges to health care systems worldwide.^1^ Hypertension, a leading cause of cardiovascular diseases (CVDs), and diabetes, a major contributor to morbidity and mortality, often co-occur, exacerbating health complications and increasing the burden on affected individuals. Hypertension affects approximately 32% of adults globally,^2^ while diabetes affects 11% of the population.^3^ Managing these conditions effectively requires comprehensive and continuous care, which can be complicated by various social determinants of health (SDOH).^4^

SDOH, including socioeconomic status, education, neighborhood and physical environment, employment, and social support networks, play a crucial role in shaping health outcomes. Individuals with lower socioeconomic status, limited education, and inadequate access to health care resources are more likely to experience poor health outcomes and have higher rates of chronic conditions.^5^ These determinants influence not only the prevalence of hypertension and diabetes but also the ability of individuals to manage these conditions effectively.^6^ Addressing SDOH is therefore essential for improving health outcomes and reducing health disparities among populations with chronic diseases.^7^ Sharing such information can significantly enhance care coordination and allow for more personalized and comprehensive treatment plans.^8^

Telehealth has emerged as a promising solution to enhance access to health care services, particularly for individuals managing multiple chronic conditions.^9^ While prior research using Health Information National Trends Survey (HINTS) has examined telehealth patterns in the broader context of CVD,^10^ the present study focuses specifically on individuals with hypertension and/or diabetes as metabolically defined subgroups, independent of CVD status. This distinction is clinically meaningful for several reasons. First, hypertension and diabetes are the 2 most prevalent chronic conditions worldwide and are frequently comanaged by primary care providers through structured protocols (eg, blood pressure monitoring, hemoglobin A1c tracking) that are particularly amenable to telehealth delivery. Second, stratifying by metabolic disease burden, comparing hypertension only, diabetes only, and both conditions, allows characterization of how telehealth use patterns and SDOH profiles differ within a clinically homogeneous high-risk group, rather than across the heterogeneous CVD spectrum. Third, this approach provides data specifically relevant to primary care telehealth program design, distinct from cardiology-focused models.^11^ Telehealth services offer several benefits, including increased accessibility, reduced travel time, and the ability to maintain regular monitoring and management of chronic conditions.^12^^,^^13^ However, the utilization and effectiveness of telehealth can be influenced by various factors, including technology access, digital literacy, and individual preferences. Understanding how each of these SDOH domains is associated with lower or higher rates of telehealth use, rather than simply observing aggregate differences between groups, is essential to designing effective, equity-focused programs.^14^ In addition, it is crucial to consider patients’ willingness to share information about their SDOH with health care providers.

Previous studies have demonstrated both the potential benefits and barriers of telehealth adoption among chronic disease populations, yet gaps remain in understanding how SDOH influence these patterns.^13^^,^^15^ This study aims to characterize and compare telehealth utilization patterns and SDOH profiles across 3 groups of adults: those with hypertension only, diabetes only, and both conditions, using nationally representative survey data. Furthermore, we aim to describe telehealth access and utilization and SDOH characteristics in individuals with hypertension, diabetes, or both conditions.^16^ We describe telehealth utilization patterns and SDOH characteristics across the 3 disease groups to identify differences that may inform future hypothesis-driven and analytic research. By describing the codistribution of SDOH factors and telehealth use across disease groups, this study provides foundational data for future studies designed to test specific hypotheses about equity in telehealth access. Understanding how SDOH impact telehealth engagement may inform targeted interventions to promote equitable access and reduce health care disparities among vulnerable populations.^17^

## Methods

### Study design

The HINTS provides data on health-related behaviors, perceptions, and access to information within the U.S. HINTS surveys are conducted annually among noninstitutionalized U.S. civilians ≥18 years of age and contain nationally representative data on awareness and use of health-related information.^18^ HINTS can shed light on the utilization patterns, effectiveness, and patient satisfaction of telehealth services among individuals with hypertension and/or diabetes.^19^^,^^20^ This study used HINTS 6 data to evaluate differences in telehealth use and related SDOH among participants with diabetes, hypertension, or both conditions.

The HINTS 6 sample comprised 6,252 respondents, with surveys conducted from March 7, 2022, to November 8, 2022. We excluded participants who had missing diabetes or hypertension status data, as well as those who reported having neither condition. We divided the sample into 3 groups: individuals with diabetes only, those with hypertension only, and those with both diabetes and hypertension. Participants’ diabetes and hypertension status were determined using 2 yes-or-no questions: “Has a doctor or other health professional ever told you that you had 1) diabetes or high blood sugar; 2) high blood pressure or hypertension?”

### Measures

We characterized the sample using participants’ self-reported sociodemographic data, including age, sex, occupational status, marital status, level of education, race, ethnicity, sexual orientation, household income, rural-urban residence status, and U.S. geographic region. Additionally, we summarized participants’ health data, encompassing self-rated health, heart and lung conditions, depression/anxiety, and cigarette use. SDOH variables included food security (ability to afford balanced meals, meal compromise due to cost), housing stability (worry about housing changes), transportation access (reliable transportation for daily needs), and willingness to share SDOH information with health care providers; each was measured using Likert-scale responses and dichotomized for analysis.

Telehealth use was measured through 14 survey questions assessing: 1) receipt of telehealth care in the past year (by video, phone, or both); 2) whether telehealth was offered as an option; 3) primary reasons for telehealth visits; 4) experiences with telehealth quality and technical issues; and 5) reasons for choosing or not choosing telehealth services.

Patient experience with telehealth was assessed via 5 domains: 1) receipt of telehealth care in the past year and modality used (video, phone, or both); 2) whether telehealth was offered as a scheduling option; 3) primary clinical reason for the most recent telehealth visit; 4) perceptions of telehealth quality, including technical difficulties, comparative care quality vs in-person visits, and privacy concerns (each measured on a 4-point Likert scale); and 5) reasons for choosing or declining telehealth. Together, these items captured both access-oriented and experience-oriented dimensions of telehealth engagement.

### Statistical analysis

HINTS utilized a complex sampling design; thus, we used weighted analyses based on the Taylor series method (linear approximation) to better estimate the variance and ensure accurate variance estimation. Data are presented as the mean ± SD for continuous variables and as count and percentage for categorical variables, reported for nonmissing data. All analyses were performed on weighted data in R Statistical Software (v4.5.1, R Core Team, 2026) with the srvyr package for complex sample weighting and the survey package for survey-appropriate statistical tests.^21^ To assess differences in participant sociodemographic, baseline health, SDOH, and telehealth use by diabetes/hypertension category, we used the Wilcoxon rank-sum test adapted for complex survey samples^22^ or the chi-squared test with Rao & Scott’s second-order correction for continuous and categorical variables, respectively.^23^

All statistical tests were 2-sided. *P* values were adjusted for multiple comparisons using the Benjamini-Hochberg procedure^24^ to control false discovery rate across multiple comparisons. The type I error rate was set to 0.05.

### Study approval

This study was deemed exempt from Institutional Review Board approval as it utilized only publicly available, deidentified data from the HINTS survey.

## Results

### Sample characteristics

Of the total HINTS 6 sample comprising 6,252 individuals, 30 respondents (weighted 0.5%) were missing diabetes status, 22 (weighted 0.4%) were missing hypertension status, and 222 (weighted 3.6%) were missing both and were therefore excluded. Additionally, 2,969 respondents (weighted 47.5%) reported having neither condition. Thus, the final sample comprised 3,009 individuals with either diabetes, hypertension, or both. The final sample comprised 1,714 individuals with hypertension only (57.0%), 317 with diabetes only (10.5%), and 978 with both conditions (32.5%). The selection process from the sample to the final analytical cohort is illustrated in Supplemental Figure 1.

### Sociodemographic and clinical characteristics

Sociodemographic characteristics across the 3 groups are presented in Table 1. Several variables showed no significant differences between groups, including sex, tobacco use, urban/rural residence, and census regions. Significant differences were observed in age (*P* < 0.001); patients with both hypertension and diabetes were the oldest (64.0 [SD = 16.3] years), followed by those with hypertension only (61.5 [SD = 17.1] years), while those with diabetes only were the youngest (57.2 [SD = 16.6] years). Regarding occupational status, respondents with only diabetes were more likely to be employed (44.9%), while those with both conditions were more likely to be retired (45.0%, *P* < 0.001). For marital status, the diabetes-only group had the highest proportion of divorced participants (22.8%) among the 3 groups, compared to 18.6% in both other groups, while having the lowest proportion of widowed participants (8.7% vs 15.0% and 17.3%). In terms of education level, respondents with both diabetes and hypertension had more individuals with high school or lower education and fewer people with a college degree or higher (*P* = 0.001). For race (*P* < 0.001) and ethnicity (*P* < 0.001), more participants with hypertension were White, and more with both conditions were Black or African American. Participants with both conditions had relatively lower household incomes (*P* < 0.001). Overall, individuals with both conditions tended to be older, retired, non-Hispanic Black or African American, and had lower education and household incomes.

**Table 1:** Characteristics of Participants

	Hypertension Only (n = 1,714)	Diabetes Only (n = 317)	Hypertension + Diabetes (n = 978)	*P* Value
Age, mean (SD), y	61.5 (17.1)	57.2 (16.6)	64.0 (16.3)	<0.001
Sex, n (%)				0.48
Male	688 (41.1)	117 (38.9)	404 (42.7)	
Female	987 (58.9)	184 (61.1)	543 (57.3)	
Occupational status, n (%)				<0.001
Employed	655 (39.0)	135 (44.9)	253 (26.5)	
Unemployed for 1 y or more	36 (2.1)	15 (5.0)	16 (1.7)	
Unemployed for < 1 y	23 (1.4)	3 (1.0)	9 (0.9)	
Homemaker	39 (2.3)	11 (3.7)	30 (3.1)	
Student	10 (0.6)	2 (0.7)	3 (0.3)	
Retired	679 (40.5)	81 (26.9)	429 (45.0)	
Disabled	95 (5.7)	27 (9.0)	98 (10.3)	
Other	11 (0.7)	1 (0.3)	7 (0.7)	
Multiple occupation statuses selected	130 (7.7)	26 (8.6)	108 (11.3)	
Marital status, n (%)				0.007
Married	749 (44.8)	129 (43.3)	407 (42.8)	
Living as married or with a romantic partner	85 (5.1)	17 (5.7)	31 (3.3)	
Divorced	311 (18.6)	68 (22.8)	177 (18.6)	
Widowed	251 (15.0)	26 (8.7)	164 (17.3)	
Separated	37 (2.2)	12 (4.0)	31 (3.3)	
Single	240 (14.3)	46 (15.4)	140 (14.7)	
Education, n (%)				0.001
High school or lower	430 (25.7)	83 (27.6)	317 (33.4)	
Post high school other than college	149 (8.9)	22 (7.3)	83 (8.7)	
Some college	374 (22.3)	62 (20.6)	223 (23.4)	
College graduate	425 (25.4)	86 (28.6)	204 (21.5)	
Postgraduate	296 (17.7)	48 (15.9)	124 (13.0)	
Ethnicity, n (%)				<0.001
Non-Hispanic White	981 (62.2)	126 (45.5)	415 (47.1)	
Non-Hispanic Black or African American	296 (18.8)	45 (16.2)	217 (24.6)	
Hispanic	196 (12.4)	80 (28.9)	178 (20.2)	
Non-Hispanic Asian	56 (3.6)	14 (5.1)	41 (4.6)	
Non-Hispanic other	47 (3.0)	12 (4.3)	31 (3.5)	
Race, n (%)				<0.001
White only	1,162 (71.3)	193 (67.7)	543 (59.8)	
Black only	336 (20.6)	55 (19.3)	257 (28.3)	
American Indian or Alaska Native only	16 (1.0)	4 (1.4)	11 (1.2)	
Multiple races	47 (2.9)	14 (4.9)	37 (4.1)	
Asian Indian only	14 (0.9)	1 (0.4)	9 (1.0)	
Chinese only	13 (0.8)	7 (2.5)	8 (0.9)	
Filipino only	12 (0.7)	4 (1.4)	16 (1.8)	
Vietnamese only	5 (0.3)	2 (0.7)	6 (0.7)	
Other Asian only	18 (1.1)	1 (0.4)	7 (0.8)	
Other Pacific Islander only	6 (0.4)	4 (1.4)	14 (1.5)	
Sexual orientation, n (%)				0.22
Heterosexual	1,543 (94.4)	268 (93.7)	850 (93.1)	
Homosexual	40 (2.4)	7 (2.4)	17 (1.9)	
Bisexual	29 (1.8)	4 (1.4)	23 (2.5)	
Else	22 (1.3)	7 (2.4)	23 (2.5)	
Income, n (%)				<0.001
Less than $20,000	283 (18.2)	57 (19.8)	229 (25.8)	
$20,000 to $34,999	209 (13.5)	47 (16.4)	135 (15.2)	
$35,000 to $49,999	209 (13.5)	40 (14.0)	149 (16.8)	
$50,000 to $74,999	270 (17.4)	51 (17.8)	141 (15.9)	
$75,000 to $99,999	208 (13.4)	27 (9.4)	99 (11.2)	
$100,000 to $199,999	278 (17.9)	44 (15.4)	99 (11.2)	
$200,000 or more	96 (6.2)	20 (7.0)	35 (3.9)	
Body mass index (BMI), mean (SD), kg/m^2^	28.9 (9.0)	29.3 (9.3)	30.2 (10.1)	0.002
General health, n (%)				<0.001
Excellent	88 (5.2)	14 (4.4)	27 (2.8)	
Very good	526 (30.9)	101 (32.1)	176 (18.2)	
Good	752 (44.2)	113 (35.9)	422 (43.6)	
Fair	288 (16.9)	76 (24.1)	279 (28.9)	
Poor	48 (2.8)	11 (3.5)	63 (6.5)	
Heart condition, n (%)	240 (14.0)	28 (8.9)	234 (24.0)	<0.001
Chronic lung disease, n (%)	282 (16.5)	53 (16.8)	204 (20.9)	0.01
Mental health problem, n (%)	448 (26.2)	98 (31.0)	298 (30.5)	0.03
Tobacco user, n (%)				0.998
Current (everyday)	142 (8.5)	27 (8.8)	82 (8.6)	
Former (some days)	60 (3.6)	10 (3.3)	33 (3.5)	
Never (not at all)	1,475 (88.0)	269 (87.9)	841 (88.0)	
Rural/urban, n (%)				0.11
Metropolitan	1,435 (83.7)	278 (87.7)	832 (85.1)	
Micropolitan	155 (9.0)	24 (7.6)	72 (7.4)	
Small town	91 (5.3)	9 (2.8)	44 (4.5)	
Rural	33 (1.9)	6 (1.9)	30 (3.1)	
Census, n (%)				0.29
Northeast	238 (13.9)	38 (12.0)	127 (13.0)	
Midwest	289 (16.9)	48 (15.1)	151 (15.4)	
South	831 (48.5)	147 (46.4)	496 (50.7)	
West	356 (20.8)	84 (26.5)	204 (20.9)	

Health characteristics also varied across the 3 respondent groups. Significant differences were noted in body mass index (*P* = 0.002), self-rated health (*P* < 0.001), presence of heart conditions (*P* < 0.001), chronic lung disease (*P* = 0.014), and mental health problems (*P* = 0.028). Participants with both hypertension and diabetes had higher body mass indexes than the other groups, while a greater proportion of them rated their general health as good or fair, and had heart disease, chronic lung disease, and mental health problems compared to the other 2 groups.

### Telehealth utilization and patient experience

**Figure 1:**
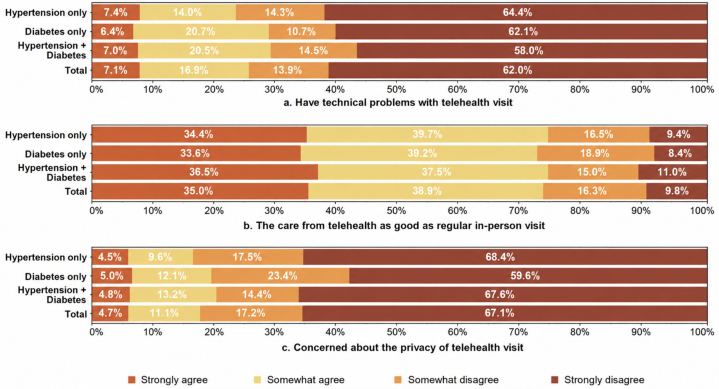
Telehealth Experiences (a) Technical problems during telehealth visits. (b) Perceived quality of telehealth care compared with in-person visits. (c) Privacy concerns about telehealth visits. Bars show weighted percentages by condition group.

Telehealth utilization patterns revealed important differences across groups. Overall, 43.9% of participants used telehealth in the past year, with convenience, provider recommendation, and infection avoidance being primary drivers (Table 2, Figure 1). We found significantly different rates of receiving a telehealth visit among participants with hypertension only, diabetes only, and both conditions (*P* = 0.001). Telehealth adoption was substantial across all groups but showed important pattern differences. While overall utilization rates were similar (43% to 48%), delivery modalities varied significantly (*P* = 0.001). Phone consultations dominated in the both-conditions group (21.4%), likely reflecting greater comfort with familiar technology among this older population. Video adoption was highest in the diabetes-only group (21.4%), potentially reflecting this group’s younger age and greater digital literacy. Utilization rates were hypertension-only 42.9% (721/1,682), diabetes-only 48.5% (150/309), and both conditions 48.0% (457/954) (Central Illustration).

**Table 2:** Characteristics of Telehealth Utilization and Patient Experiences

	Hypertension Only (n = 1,714)	Diabetes Only (n = 317)	Hypertension + Diabetes (n = 978)	*P* Value
Received telehealth care, n (%)				0.001
Yes, by video	298 (17.7)	66 (21.4)	160 (16.8)	
Yes, by phone call (no video)	248 (14.7)	56 (18.1)	204 (21.4)	
Yes, some video some phone call	175 (10.4)	28 (9.1)	93 (9.8)	
No telehealth visits	961 (57.1)	159 (51.5)	496 (52.0)	
Offered telehealth option, n (%)				0.03
Yes	161 (17.4)	20 (13.2)	101 (21.5)	
No	636 (68.8)	114 (75.0)	323 (68.9)	
Did not try	127 (13.7)	18 (11.8)	45 (9.6)	
Primary reason for telehealth, n (%)				0.002
Annual visit	161 (23.5)	29 (20.0)	100 (23.8)	
Minor illness	148 (21.6)	23 (15.9)	53 (12.6)	
Manage chronic health	206 (30.1)	50 (34.5)	171 (40.7)	
Medical emergency	13 (1.9)	3 (2.1)	9 (2.1)	
Mental health	68 (9.9)	20 (13.8)	28 (6.7)	
Other	88 (12.9)	20 (13.8)	59 (14.0)	
Regarding telehealth visits, how much do you agree or disagree n (%)				
a. I had technical problems with telehealth				0.10
Strongly agree	49 (7.4)	9 (6.4)	28 (7.0)	
Somewhat agree	93 (14.0)	29 (20.7)	82 (20.5)	
Somewhat disagree	95 (14.3)	15 (10.7)	58 (14.5)	
Strongly disagree	428 (64.4)	87 (62.1)	232 (58.0)	
b. Telehealth was as good care as in-person visits				0.84
Strongly agree	234 (34.4)	48 (33.6)	153 (36.5)	
Somewhat agree	270 (39.7)	56 (39.2)	157 (37.5)	
Somewhat disagree	112 (16.5)	27 (18.9)	63 (15.0)	
Strongly disagree	64 (9.4)	12 (8.4)	46 (11.0)	
c. I had privacy concerns about telehealth				0.16
Strongly agree	30 (4.5)	7 (5.0)	19 (4.8)	
Somewhat agree	64 (9.6)	17 (12.1)	52 (13.2)	
Somewhat disagree	116 (17.5)	33 (23.4)	57 (14.4)	
Strongly disagree	454 (68.4)	84 (59.6)	267 (67.6)

**Central Illustration:**
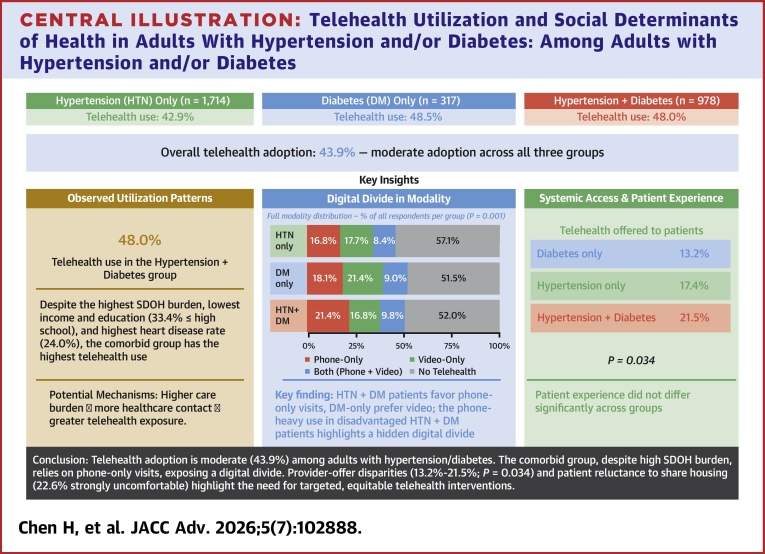
**Telehealth Utilization and Social Determinants of Health in Adults With Hypertension and/or Diabetes: Among Adults with Hypertension and/or Diabetes** [Population]: 3,009 U.S. adults with hypertension only, diabetes only, or both conditions (HINTS 6, 2022). [Key findings]: Despite carrying the greatest SDOH burden, adults with both conditions had the highest telehealth use (48.0%). No significant differences in technical difficulties or care quality across groups. Equitable telehealth access requires addressing underlying SDOH barriers. HINTS = Health Information National Trends Survey; SDOH = Social Determinants of Health.

Regarding being offered a telehealth visit option, feedback from the 3 groups was also significantly diverse. Telehealth was offered as a scheduling option to only 17.4% of participants with hypertension, 13.2% with diabetes, and 21.5% with both conditions (*P* = 0.034), suggesting that provider-initiated telehealth referral remains uncommon across all groups. The primary reason selected for a recent telehealth visit was significantly different among the groups (*P* = 0.002). For participants with hypertension, 206 (30.1%) were for managing chronic health, followed by 161 (23.5%) for annual visits, 148 (21.6%) for minor illness, 88 (12.9%) for other reasons, 68 (9.9%) for mental health, and 13 (1.9%) for medical emergency. For participants with diabetes, 50 (34.5%) were for managing chronic health, followed by 29 (20.0%) for annual visits, 23 (15.9%) for minor illness, 20 (13.8%) for mental health and other reasons, and 3 (2.1%) for medical emergency. For participants with both hypertension and diabetes, 171 (40.7%) were for managing chronic health, 100 (23.8%) for annual visits, 59 (14.0%) for other reasons, 53 (12.6%) for minor illness, 28 (6.7%) for mental health, and 9 (2.1%) for medical emergency. Apparently, most patients utilize telehealth for their long-term health conditions and chronic diseases, instead of emergencies requiring urgent and in-person treatment (Table 2).

We also noted that most individuals who experienced a telehealth visit had few technical obstacles, with overall 62.0% strongly disagreeing and 13.9% somewhat disagreeing to having technical trouble with a telehealth visit. Most individuals agreed that the care from telehealth was as good as an in-person visit with overall 35.0% strongly agreeing and 38.9% somewhat agreeing. Also, most (67.1%) did not have privacy concerns with telehealth visits (Figure 1). However, no statistically significant differences among the groups on their thoughts and experiences with a telehealth visit were found, particularly with having technical problems with telehealth (*P* = 0.10), regarding telehealth as good as an in-person visit (*P* = 0.84), and having privacy concerns with telehealth (*P* = 0.16) (Table 2).

Similarly, we analyzed reasons for selecting or declining telehealth visits. We found that most individuals did not specify their reasons for not selecting a telehealth visit. Of the patients who responded to the survey, 90.3% of patients in general reflected that they preferred an in-person visit, whereas 80.3% of patients were not concerned about the privacy of telehealth visits. Furthermore, only 27.6% of patients thought that telehealth would be difficult to use.

Participants’ reasons for choosing telehealth were presented in Table 3. Most (73.2%) agreed that they chose to have a telehealth visit because of the recommendation by their health care provider, while only 26.4% were asking for advice on whether in-person care is needed. About half (49.2%) of individuals reported that they wanted to avoid potential infection in hospital, and 57.7% of them believed that it was more convenient than going to the doctor, saving travel and waiting time. However, no statistically significant differences were found among the 3 groups with regard to these questions. The only difference in opinions was about whether family or other caregivers could be included in the appointment (*P* = 0.022), with 25.9% of participants having both hypertension and diabetes placing greater emphasis on this factor compared to hypertension (19.5%) and diabetes alone (17.8%).

**Table 3:** Reasons for Not Choosing or Choosing a Telehealth Visit

	Hypertension Only (n = 1,714)	Diabetes Only (n = 317)	Hypertension + Diabetes (n = 978)	*P* Value
Did you choose not to participate in a telehealth visit because you				
a. Preferred to have the appointment(s) in person?				0.30
Yes	147 (89.6)	21 (100.0)	94 (89.5)	
No	17 (10.4)	0 (0.0)	11 (10.5)	
b. Were concerned about the privacy of telehealth visits?				0.77
Yes	30 (18.3)	4 (19.0)	23 (21.9)	
No	134 (81.7)	17 (81.0)	82 (78.1)	
c. Thought the technology would be difficult to use?				0.41
Yes	41 (25.3)	5 (23.8)	34 (32.4)	
No	121 (74.7)	16 (76.2)	71 (67.6)	
Why did you choose a telehealth visit(s)?				
a. The health care provider recommended or required the use of telehealth for the visit.				0.051
Yes	518 (75.8)	108 (73.0)	294 (69.2)	
No	165 (24.2)	40 (27.0)	131 (30.8)	
b. You wanted advice about whether you needed in-person medical care.				0.19
Yes	168 (24.7)	46 (31.7)	114 (27.3)	
No	513 (75.3)	99 (68.3)	303 (72.7)	
c. You wanted to avoid possible infection at the doctor’s office or hospital (eg, COVID-19 or flu).				0.34
Yes	334 (48.7)	66 (44.9)	220 (51.6)	
No	352 (51.3)	81 (55.1)	206 (48.4)	
d. It was more convenient than going to the doctor (eg, less travel or waiting time).				0.35
Yes	404 (59.2)	86 (58.9)	231 (54.9)	
No	278 (40.8)	60 (41.1)	190 (45.1)	
e. You could include family or other caregivers in your appointment.				0.02
Yes	133 (19.5)	26 (17.8)	109 (25.9)	
No	549 (80.5)	120 (82.2)	312 (74.1)	

### Social determinants of health

**Figure 2:**
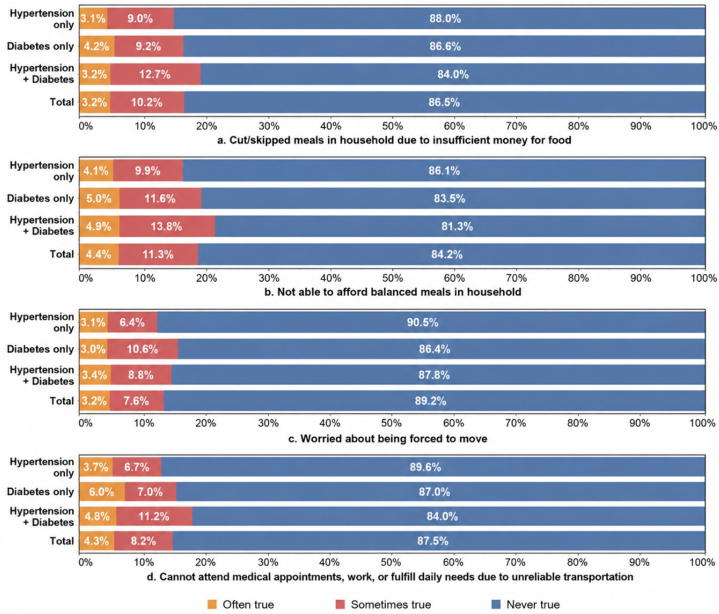
Social Determinants of Health Challenges (a) Meals cut or skipped because of insufficient money for food. (b) Inability to afford balanced meals. (c) Worry about being forced to move. (D) Inability to attend medical appointments, work, or daily activities because of unreliable transportation. Bars show weighted percentages by condition group.

**Figure 3:**
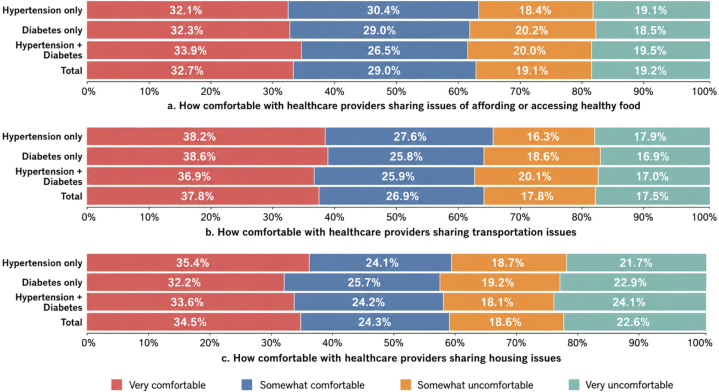
Willingness to Share Social Determinants of Health Information (a) Comfort with health care providers sharing information about food-related issues. (b) Comfort with health care providers sharing information about transportation-related issues. (c) Comfort with health care providers sharing information about housing-related issues. Bars show weighted percentages by condition group.

SDOH characteristics across groups are depicted in Figures 2 and 3. Concerns such as food insecurity, affordability of balanced meals, worries about relocation, and transportation issues were prevalent across all groups. Figure 2 shows that 86.5% of people in total did not need to compromise on meals, 84.2% could afford balanced nutrition, and 87.5% did not face challenges in accessing reliable transportation for their daily needs, although the situation of participants with both hypertension and diabetes was slightly worse than the other 2 groups. Only 10.8% of participants overall reported concerns about housing or relocation; however, this was highest in the diabetes-only group. Overall, people with only hypertension had the best situation among the groups, while people with both hypertension and diabetes faced more difficulties.

Opinions on whether participants would be willing to allow health care providers to share their related information on food, transportation, and housing for treatment purposes are shown in Figure 3 and were relatively evenly distributed and had little difference among the 3 groups. Concerning the food issues, 32.7% of people were very supportive and 29.0% felt comfortable about it, while the rest felt a bit offensive. For the transportation issue, people were slightly more comfortable sharing the information, with 37.8% feeling completely comfortable and 26.9% comfortable. However, 22.6% of people felt strongly uncomfortable and 18.6% felt uncomfortable if their housing issues were shared. In general, people tended to be more concerned about the privacy of their housing conditions.

## Discussion

The findings from our analysis provide valuable insights into the utilization of telehealth among individuals managing hypertension and/or diabetes. By characterizing individuals with hypertension and/or diabetes according to their sociodemographic characteristics, health status, and SDOH factors, we identified important patterns in telehealth utilization across these groups. Our analysis revealed no significant differences in telehealth experiences were observed across condition groups (technical problems *P* = 0.10, care quality *P* = 0.84, privacy concerns *P* = 0.16), which is consistent with telehealth's broad use in this population. The substantial adoption rate of 43.9% among this chronic disease population, combined with high satisfaction ratings (73.9% rating telehealth as good as in-person visits), demonstrates its effectiveness for this population. This study's strengths include the use of nationally representative data, comprehensive assessment of SDOH factors, and focus on multiple chronic conditions simultaneously. Our work addresses gaps in understanding telehealth utilization patterns across different chronic disease combinations.

### Privacy considerations

Privacy concerns were minimal and did not significantly differ across groups (67.1% reporting no concerns; *P* = 0.16). While this finding may seem to contradict commonly cited barriers to telehealth adoption, it is consistent with studies showing that patients with established health care relationships are less likely to cite privacy as a deterrent.^25^^,^^26^ Telehealth providers should nonetheless maintain transparent data-sharing policies and offer patients meaningful control over their information,^27^ as building and sustaining trust is an ongoing process.^28^ The nonsignificant finding should not be interpreted as an absence of privacy concerns at the population level, but rather as an absence of differential concern by disease group in this sample.^29^

### Health equity and telehealth access

Participants with both conditions—who had the highest telehealth utilization (48.0%)—were paradoxically those with the greatest socioeconomic challenges (lower income, education, and multiple comorbidities). This suggests existing health care relationships may overcome traditional digital divide barriers, though significant (*P* = 0.034) disparities in being offered telehealth options in participants with both hypertension and diabetes (21.5%) compared to diabetes (13.2%) and hypertension (17.4%) only indicate systemic access issues remain. Health care provider recommendation was the most frequently cited reason for telehealth use across all groups, co-occurring with high utilization rates regardless of SDOH burden.^30^^,^^31^ While convenience and health care provider recommendations are significant drivers of telehealth adoption, efforts should also be directed toward addressing systemic barriers and disparities in access.^32^ Initiatives to improve digital literacy, expand broadband infrastructure, and enhance reimbursement policies for telehealth services can help mitigate these barriers and ensure equitable access to care.^17^^,^^33^ Participants' willingness to share information on food, transportation, and housing with health care providers varied, with the most concern expressed about sharing housing information. Health care providers should ensure that data sharing is conducted transparently and securely to build trust with patients and encourage information sharing that can enhance care coordination and support.

The finding that the comorbid group exhibited both the greatest SDOH burden and the highest telehealth utilization rate (48.0%) warrants careful interpretation. This apparent paradox may reflect at least 2 distinct mechanisms. First, individuals with multiple chronic conditions engage more frequently with the health care system overall, increasing the probability of exposure to telehealth offers regardless of socioeconomic barriers—a “care burden effect.” Second, and critically, our data show that phone-only consultations were disproportionately prevalent in the comorbid group (21.4% phone-only vs 16.8% video-only), compared to the diabetes-only group (18.1% phone vs 21.4% video). This suggests that the higher utilization in the comorbid group may partly reflect reliance on audio-only calls as a low-technology necessity for an older, lower-income population with limited digital literacy or broadband access, rather than equitable adoption of full-featured telehealth services. Thus, while aggregate utilization rates appear similar across groups, the modality data reveal a meaningful digital divide: the most socioeconomically disadvantaged patients are accessing telehealth through its least feature-rich form. Future work employing multivariable modeling should formally test whether SDOH factors moderate the association between disease burden and telehealth modality choice, and whether phone-only visits achieve comparable clinical outcomes to video-based care in this population.^34^

### Patients managing comorbidities

The effective management of multiple chronic conditions through telehealth presents a promising avenue for enhancing patient outcomes.^19^ Participants with both hypertension and diabetes demonstrated successful telehealth adoption (48.0%) despite facing the greatest complexity. Our analysis revealed participants with both hypertension and diabetes were significantly older compared to participants with hypertension only and diabetes only (64.0 vs 61.5 vs 57.2 years, respectively; *P* < 0.001), had more comorbidities such as heart disease (24.0% vs 14.0% vs 8.9%, respectively; *P* < 0.001), and lower socioeconomic status, yet maintained utilization rates comparable to less complex patients. Importantly, this combined group showed distinct usage patterns: they were most likely to use telehealth for chronic disease management compared to individuals with diabetes and hypertension only (40.7% vs 34.5% vs 30.1%, respectively; *P* = 0.002) and more likely to include family caregivers in their appointment (25.9% vs 17.8% vs 19.5%, respectively; *P* = 0.022), suggesting telehealth may be particularly valuable for complex patients requiring ongoing monitoring and family support. While technical difficulties were not significantly different across groups (*P* = 0.10), this result may reflect insufficient statistical power to detect differences <10% that may be clinically relevant rather than a true absence of technology barriers among older, lower-income comorbid patients.

Our results indicate that telehealth services are frequently used for managing long-term health conditions and chronic diseases rather than for urgent care needs.^35^ This is particularly important for individuals with both hypertension and diabetes, who require continuous monitoring and management of their conditions. Telehealth may support hypertension and diabetes management by facilitating regular monitoring and remote consultations, reducing the need for in-person visits, and easing travel burdens, especially for rural or underserved populations; however, whether it does so effectively for this population requires prospective or causal study designs.^36^ Improved accessibility through telehealth can enhance treatment adherence and overall disease management.^37^ However, accessing and adopting telehealth services may be more challenging for patients managing multiple chronic conditions.^38^ The difference in telehealth use among these groups stems from various factors. Individuals managing a single chronic condition, like hypertension or diabetes, may find telehealth convenient for tasks such as monitoring health metrics and managing medications.^16^^,^^17^^,^^39^ They face fewer competing health care needs and logistical hurdles, making it easier to integrate telehealth into their care routine.^40^

On the other hand, patients with both diabetes and hypertension face a heavier care burden, requiring more frequent interactions with health care providers and specialized treatments.^41^^,^^42^ The complexity of their health care needs may hinder the effective incorporation of telehealth into their care plan, with challenges like accessing necessary technology and coordinating virtual appointments with multiple providers.^43^ Additionally, those contending with multiple chronic conditions may encounter barriers related to digital literacy or socioeconomic status, such as limited access to reliable internet or devices.^44^^,^^45^ These disparities in technology access disproportionately affect certain patient groups, exacerbating telehealth utilization inequalities.^46^

To tackle these disparities, health care systems and policymakers should implement targeted interventions to support patients managing multiple chronic conditions in accessing and utilizing telehealth services effectively.^17^^,^^47^ This may involve providing digital literacy training, offering loaner devices or internet connectivity solutions, and collaborating with community organizations to address SDOH impacting telehealth access.^48^ By addressing these systemic barriers, health care providers can ensure equitable access to the convenience and accessibility of telehealth services for all patients, regardless of their health status or socioeconomic circumstances.^17^^,^^49^

### Addressing social determinants of health challenges

Our SDOH analysis revealed a clear gradient of challenges corresponding to disease complexity. Food security issues were most prevalent among participants with both conditions: 13.5% could not afford balanced meals compared to better outcomes in single-condition groups. Transportation barriers followed a similar pattern, with the both-conditions group reporting the highest rates of transportation difficulties for daily needs. Housing stability concerns were highest in the diabetes-only group (10.8% worried about housing changes), possibly reflecting this group's younger age and economic instability. These findings describe a pattern in which greater SDOH burden co-occurs with greater telehealth use, particularly in the comorbid group; further research is needed to determine whether SDOH factors directly affect telehealth access.^19^ Factors like income, education, housing stability, transportation, and food security all play a significant role in a person's ability to use telehealth effectively.^16^^,^^49^

By addressing these underlying SDOH, health care providers and policymakers can take significant strides toward ensuring equitable access to telehealth services for all individuals, regardless of their socioeconomic circumstances.^17^^,^^47^ This may involve implementing targeted interventions such as providing subsidies for internet access, offering transportation assistance, or partnering with community organizations to address food insecurity.^50^ Addressing these disparities is crucial to ensuring equitable access to telehealth services and reducing health care inequities.^51^

Despite these SDOH challenges, telehealth utilization remained high across all groups, suggesting telehealth may actually help overcome some traditional access barriers. However, willingness to share SDOH information varied, with housing information being the most sensitive (22.6% strongly uncomfortable sharing), potentially limiting providers' ability to address social needs during telehealth visits. The persistence of high telehealth utilization despite SDOH challenges suggests these services may be particularly valuable for populations facing traditional health care access barriers, though targeted interventions addressing specific SDOH factors may further improve outcomes.^52^

Moving forward, it is important for future studies to investigate the long-term effects of using telehealth on health care outcomes for people managing hypertension and diabetes.^53^ Additionally, further investigation into the SDOH factors driving the observed disparities is warranted.^54^ This includes developing tailored interventions to enhance telehealth engagement and improve outcomes across diverse patient demographics.^17^

### Study Limitations

This study has several limitations. The cross-sectional design limits the ability to establish causality between the observed characteristics and health outcomes. The data in this study rely on participants' self-reported responses, which could introduce biases. Recall bias may occur if participants do not accurately remember or report their behaviors and experiences. Social desirability bias is also a concern, as participants might provide responses they think are more socially acceptable, rather than reflecting their true experiences.^55^ Additionally, the study sample might not fully represent the broader population, potentially limiting how widely we can apply our findings.^56^, ^57^, ^58^

## Conclusions

This study highlights the substantial adoption of telehealth among individuals managing hypertension and diabetes, underscoring its potential to revolutionize chronic disease management. This study demonstrated similar telehealth utilization rates across chronic disease groups despite different demographic profiles, significant SDOH challenges among individuals with multiple conditions, and disparities in access to telehealth options and caregiver involvement preferences. These findings suggest addressing privacy concerns and leveraging health care provider recommendations are crucial for fostering wider telehealth adoption. Future research should focus on the long-term impacts of telehealth and further investigate SDOH factors to develop tailored interventions that enhance engagement and improve outcomes across diverse patient populations. By prioritizing these areas, we can maximize telehealth's benefits, ensuring it serves as a transformative tool in health care delivery.

## Funding support and author disclosures

The authors have reported that they have no relationships relevant to the contents of this paper to disclose.
