# Supplementary figures and images for: Telehealth Utilization and Patient Experiences: Patterns of Social Determinants of Health Among Individuals With Hypertension and Diabetes
Source: JACC Adv. 2026 Jul 22;5(7):102888. doi: 10.1016/j.jacadv.2026.102888 (PMC13400121; doi:10.1016/j.jacadv.2026.102888)

## Supplemental Figure

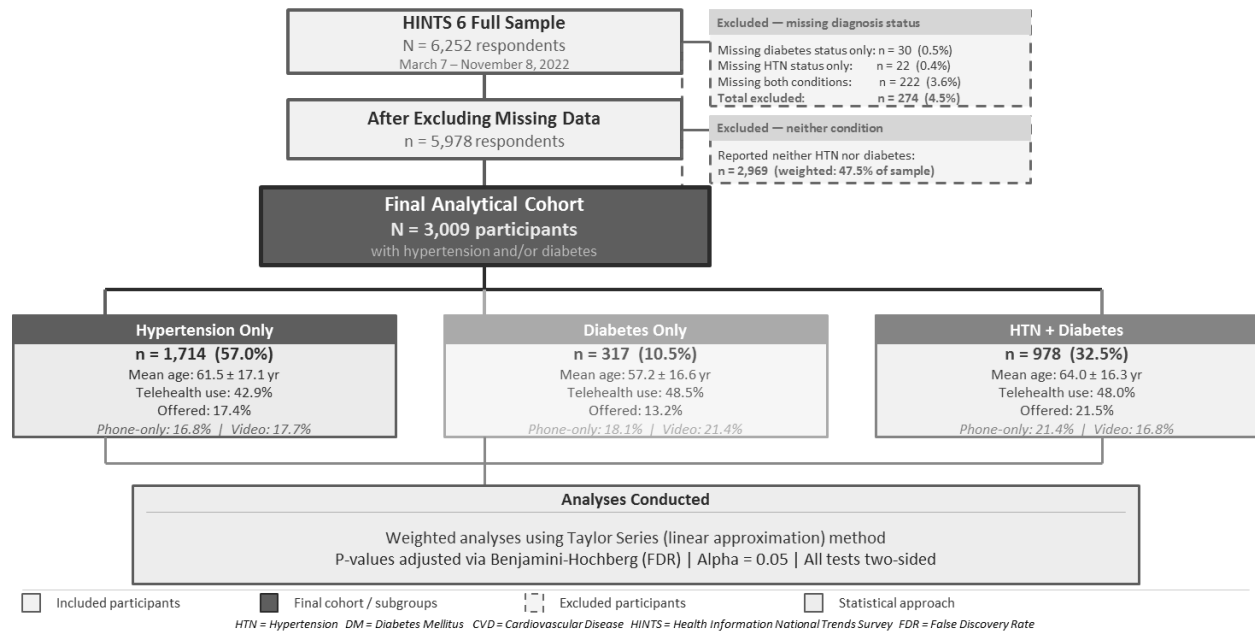

**Figure 1.** Flow chart of the cohort

Supplement: Supplemental_Material [file mmc1.pdf]
